# Supplementary material for: Impact of visuomotor feedback on the embodiment of virtual hands detached from the body
Source: Sci Rep. 2020 Dec 30;10:22427. doi: 10.1038/s41598-020-79255-5 (PMC7773737; doi:10.1038/s41598-020-79255-5)
Supplement: Supplementary file 3 — Supplementary Information 2. [file 41598_2020_79255_MOESM3_ESM.docx]

Supplementary Materials

Impact of Visuomotor Feedback on the Embodiment of Virtual Hands Detached from the Body

Sofia Seinfeld^1^ & Jörg Müller^1^

^1^Institute of Computer Science, University of Bayreuth, Bayreuth, Germany

## **Skin Conductance Response (SCR) data recording**

SCR was measured using a ProComp Infiniti Encoder (Thought Technologies Ltd) connected to a PC through a serial port and based on the placement of two galvanic skin response sensors (i.e., SA9309M bipolar electrodes) on the participants’ middle and index of the right hand. The signal was sampled at 256Hz and low-pass filtered with a cut-off frequency of 1Hz. The data was pre-processed and analyzed using Matlab. Before the analysis, SCR data was visually inspected to detect possible undesired noise and artefacts due to motion. The experiment was programmed to send an event marker to the SC signal as soon as the virtual threat started.

## **Pre-processing and analysis of threat-related SCR**

Our main analysis was based on baseline-to-peak raw skin conductance responses by computing the maximum skin conductance value during a 10sec window after the occurrence of the virtual threat (i.e., harm by a virtual shuriken), in relation to a baseline taken 0.30sec before the occurrence of the threat. This specific parameter was selected for analysis, since it has been frequently used by past research to analyze SCR related to a threat or painful stimuli^24,36,37^. The decision of analyzing a 10 sec time window was based on the visual inspection of the data, which indicated that at 10 sec after the virtual threat onset the averaged skin conductance signal already reached its peak responses (**Figure 4**). Finally, we also calculated the standard deviations and slope of the SC signal for additional analysis.

As in the case of motor performance, threat-related SCR were analyzed using mixed-design ANOVAs with SPSS version 24. In this analysis, the factor *Body Visual Continuity* was included as a within-group factor and the factors *Awareness* and *Order* were set as the between-group factors. The factor *Awareness* was included in the analysis to control for the potential influence of participants consciously noticing or not the experimental manipulation (i.e., visual continuity of the body). Moreover, despite the use of a full counterbalanced design, we further included the factor *Order* in this analysis to thoroughly control for physiological habituation effects related to the repeated exposure to the virtual threat^38^ and to control for potential learning effects (i.e., training) in the motor task^39^. The residual errors of the ANOVA analysis were tested for normality using Shapiro-Wilk tests. Significance of results was calculated with a 95% confidence level.

## **Threat-related SCR Results**

As shown in **Figure 1S**, most participants had a pronounced increase in their skin conductance response after being virtually threatened by a shuriken. Below we describe the results of the ANOVAs carried out to analyze threat-related SCR in a 10 sec time window after the virtual threat onset. The analysis was carried out in a total sample of n=30, since the data of one participant was not correctly logged.

In a 10 sec time window after the virtual threat onset, the main effects of *Body Visual Continuity* (F(1,26)=0.50, p=0.49, partial η2=0.02), *Order* (F(1,26)=0.06, p=0.81, partial η2=0.01), or *Awareness* (F(1,26)=1.43, p=0.24, partial η2=0.05) on SCR were not significant. Moreover, no significant interaction effects were found between *Body Visual Continuity***Order* (F(1,26)=2.66, p=0.12, partial η2=0.09) or between *Body Visual Continuity*Awareness* (F(1, 26)=2.79, p=0.11, partial η2=0.10). Therefore, the SCR percentage of amplitude change in response to a virtual threat did not differ between the *Connected* (Mean=39.09, SD=31.83) and *Disconnected* hands (Mean=40.78, SD=29.30) in a 10 sec time window. The ANOVA residual errors were normally distributed.

We additionally also run the same analysis using the mean SCR standard deviations and slope coefficients as the dependent variables, finding no significant differences.


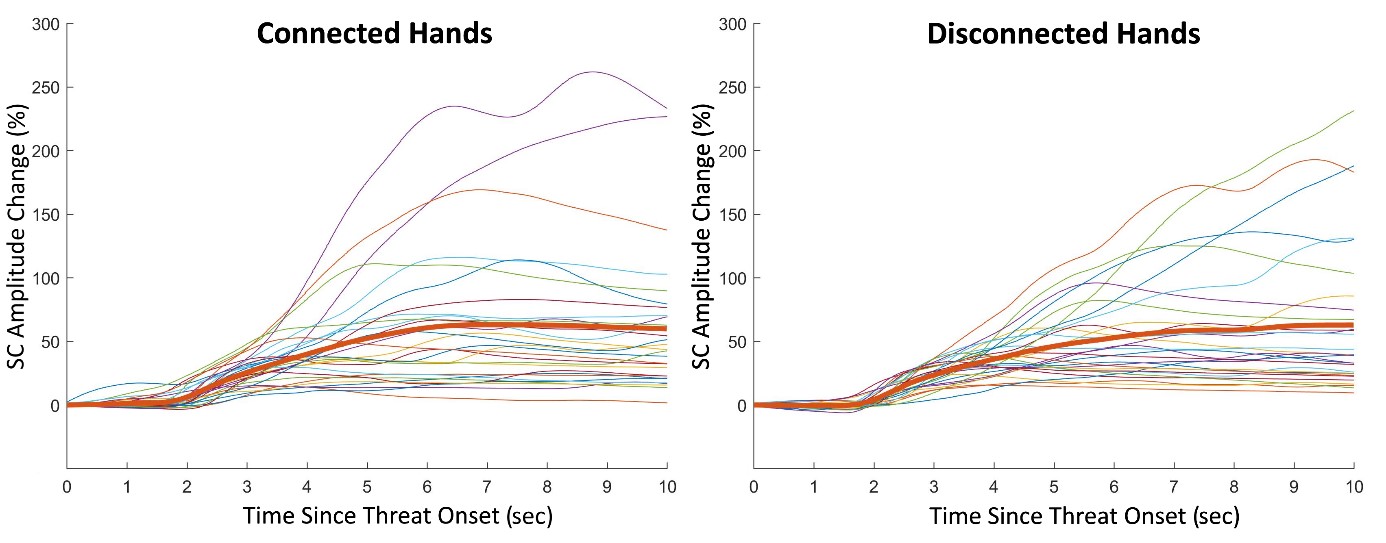


Figure 1S. Percentage of change in skin conductance (SC) amplitude 10 seconds after the virtual threat onset (i.e., virtual shuriken harming the hand). The red thick line represents the mean values of all participants, while the thinner lines represent individual values.
